# Homemade Chemical Bomb Incidents — 15 States, 2003–2011

**Published:** 2013-06-21

**Authors:** Sherrie L. Strain, Wanda Lizak Welles, Theodore C. Larson, Maureen F. Orr, Jennifer Wu, D. Kevin Horton

**Affiliations:** Bur of Toxic Substance Assessment, New York State Dept of Health; Div of Toxicology and Human Health Sciences, Agency for Toxic Substances and Disease Registry

Homemade chemical bombs (HCBs) are made from commonly found chemicals. The volume of news reports of HCB explosions suggests they are not uncommon. To determine the number of events involving HCBs in the United States and describe the factors associated with them, the Agency for Toxic Substances and Disease Registry (ATSDR) analyzed data from its surveillance system that tracks spills and leaks of hazardous substances. This report describes the results of that analysis, which indicated that, during 2003–2011, a total of 134 events involving HCBs were reported from 15 states. Among those events, 21 (16%) resulted in adverse health effects (i.e., respiratory symptoms, burns, and skin irritation) for 53 persons. The majority (35 [66%]) of these persons were youths. HCBs are hazardous and especially dangerous if detonated in public areas. Increasing awareness of HCBs and their dangers (particularly during summer months) among first-responders, parents, school staff members and others who work with youths might help reduce injuries associated with HCBs.

HCBs are explosives made from readily available chemicals, and instructions for making them are accessible on the Internet. Typically, HCB ingredients are combined in a container, such as a soft drink bottle, which is then sealed and shaken. HCBs explode when the pressure from gases produced by the chemical reaction ruptures the container. The resulting explosion can be unpredictable in both timing and magnitude. Potential hazards include exposure to the blast, shrapnel, and hazardous substances. This report uses data from the ATSDR Hazardous Substances Emergency Events Surveillance (HSEES) system and the National Toxic Substance Incidents Program (NTSIP), which replaced HSEES in 2010 (1), and updates a previous report (2). ATSDR has maintained a state-based surveillance program since 1990. The purpose of these surveillance systems is to track the public health consequences (e.g., morbidity and mortality) from acute toxic substance releases.

Incident records from states that participated in the surveillance program for at least 3 years during 2003–2011 were searched for the keywords “bottle,” “bomb,” or “homemade” in database fields that contain a synopsis of the event and health department comments. The resulting records were then reviewed, and those containing the keywords but not involving an HCB were excluded. Exclusions included events involving pesticide “bug bombs” or chemical bottles inadvertently broken during shipping. Events involving commercial or other, improvised explosives (e.g., pipe bombs) also were excluded.

During 2003–2011, a total of 134 events involving an HCB (0.2% of all HSEES/NTSIP events for the same period) were detected (Table). The number of participating states varied during the reporting period from 15 in 2003 to six in 2011. Notably, New York, Wisconsin, and Minnesota reported 77% of the events. Following are three illustrative case reports on HCB incidents with injuries.

## Case Reports

### Incident A

A high school janitor found students mixing calcium hypochlorite and other chemicals in a bottle. The janitor seized the bottle, which exploded, releasing chlorine gas. The janitor became ill and vomited, and 12 students and three school workers were treated for respiratory problems. Approximately 1,640 persons were evacuated for 5 hours while a hazardous materials team cleaned and ventilated the school.

### Incident B

Two adults were preparing an HCB from hydrochloric acid and aluminum when it prematurely exploded. First responders found one adult unconscious, and both adults sustained physical trauma, respiratory symptoms, and chemical burns. They were treated at a local hospital.

### Incident C

An adult picked up an HCB he found outside his home. Without warning, the HCB exploded in his hand. The man sustained trauma and chemical burns to his hand and chest.

## Epidemiologic Findings

Twenty-one (16%) of the 134 events identified for the period 2003–2011 resulted in 53 persons with adverse health effects. Thirteen events had one injured person, three events had two, two events had four, two events had five, and one event (incident A) had 16 injured persons. The proportion of HCB events resulting in adverse health effects was 45% greater than that of all other HSEES/NTSIP events during the same period (16% versus 11%). The majority of injured persons were male (29 [55%]); eight (15%) were female, and sex was unknown for 16 (30%). Thirty-five injured persons (66%) were youths; 17 (32%) were adults, and age was unknown for one. Twenty-one injured persons (40%) were students at school; 20 (38%) were members of the public; seven (13%) were employees at the site of the incident, and five (9%) were police officers.

Several injured persons reported more than one adverse health effect; the total number of reported adverse health effects was 62. Respiratory symptoms were most common (26 [42%]), followed by burns (14 [23%]), skin irritation (13 [21%]), and physical trauma (six [10%]). A total of 29 injured persons (55%) were treated on the scene. Fifteen (28%) were treated at the hospital and released; five (9%) were treated at the hospital and admitted; three (6%) had untreated injuries; and treatment data were missing for one. Among all 53 injured persons, 21 (40%) required decontamination at the scene or at a medical facility. No fatalities occurred in any of these events. In two events, first responders who were not injured (eight and four, respectively) were decontaminated at the scene.

The most common chemicals in these events were acids or bases mixed with a metal. Commercial household products, such as toilet bowl cleaners containing sulfuric or hydrochloric acid or drain openers containing sodium hydroxide or potassium hydroxide, were the most common sources of acids and bases. Aluminum was the most common metal. One event involved carbon dioxide (i.e., dry ice) as the main bomb ingredient.

Most HCB explosions were reported in schools, mail boxes, and residential backyards. Facility evacuations ordered by an official occurred in 17 (13%) of the 134 events. Some evacuations resulted in significant disruptions; four events, all in schools, involved evacuations of 600 or more persons for up to 8 hours.

A total of 48 events (36%) occurred within a quarter mile of a school. Summer was the season with the greatest number of events 49 [37%]), followed by fall (34 [25%]), spring (28 [21%]), and winter (23 [17%]).

What is already known on this topic?

Homemade chemical bombs (HCBs) are made from readily available chemicals. Instructions for making HCBs are accessible on the Internet. Potential hazards from HCB explosions include exposure to the blast, shrapnel, and hazardous substances. Reports of HCB explosions are not uncommon in the United States news media; however, few data on them exist in the scientific literature.

What is added by this report?

Surveillance data from 15 states during 2003–2011 identified 134 events involving HCBs. Twenty-one (16%) events resulted in 53 injured persons with adverse health effects. The majority of these injured persons were youths with health effects associated with exposure to HCB contents, including respiratory symptoms, burns, and skin irritation.

What are the implications for public health practice?

HCBs are hazardous and especially dangerous if detonated in public areas such as schools. It is important for parents, school staff members, and law enforcement to be aware of the potential hazards of HCBs and how to respond if an HCB is found.

### Editorial Note

For the period January 1996–March 2003, ATSDR reported 29 events involving HCBs (2). Standardized by state-surveillance year, the rate of HCB events in that report was 0.21 (29 events per 137 state-years). In the present report, that rate was 1.26 (134 events per 106 state-years), suggesting an increase in HCB events. This increase might be the result of greater availability of materials and Internet instructions for making HCBs, the ease with which they are made, and copycatting inspired by other incidents. However, improved HSEES/NTSIP event ascertainment also might have contributed to this increase. Participating states rely heavily on media news reports for HCB event ascertainment; for 50% of HCB events the primary reporting source was the media, versus only 5% for other hazardous substance events. In addition, HCB incidents are more likely to appear in news reports if they involve injured persons or property damage. Thus, the number of HCB incidents in HSEES/NTSIP likely is less than the actual number of events, and HCB incidents with injured persons or property damage might be overrepresented.

Although unlikely to have the injury patterns associated with high-order explosive denotations, HCB explosions have the potential to result in serious injury. In addition to blast-induced trauma, injured persons can be exposed to the chemicals released from the HCB. The most common injuries reported were respiratory symptoms, burns, and skin irritation, and these are consistent with exposure to the acids or bases frequently used in these devices. Acid and base solutions are corrosive to skin and other tissues, and both form fumes that can irritate respiratory tissues when inhaled. Symptoms associated with inhalation of fumes of acids or bases include irritation of the nose, throat, and larynx; cough; and pulmonary edema (3).

The findings in this report are subject to at least three limitations. First, searching the HSEES/NTSIP databases might not capture all events involving HCBs. Second, variability in the number of HCB incidents by state might be explained by differences in state surveillance sources or by copycatting inspired by other incidents. Finally, the number of participating states is limited, and their data might not be representative of the entire United States.

These data indicate that the majority of HCB-injured persons were youths or young adults. Consequently, it is important for parents, school staff members, and law enforcement to be aware of the potential hazards of HCBs and how to respond if an HCB is found. If a suspected or actual HCB is discovered, the surrounding area should be isolated until the situation is assessed by authorities (4). Only trained bomb squad personnel should approach, handle, or attempt to neutralize these devices (2). Persons whose clothing is contaminated with the contents of a bomb, whether as a result of the container bursting or from leakage, should remove contaminated clothing immediately (2,5). If the contents of a bomb come in contact with skin, the affected area should be rinsed with large amounts of water for 3–5 minutes (5). If severe adverse health effects (e.g., trauma, chemical burns, or respiratory irritation) occur, medical attention should be sought immediately.

## Figures and Tables

**TABLE:** Number and annualized incidence of events involving homemade chemical bombs, years of state participation, and annualized incidence by state — Hazardous Substances Emergency Events Surveillance/National Toxic Substance Incidents Program (HSEES/NTSIP), 15 states, 2003–2011

State	Period of state participation	No.	No. of years of state participation	Annualized incidence
**Total**	**—**	**134**	**106**	**1.26** *
Colorado	2003–2009	0	7	0.00
Florida	2005–2009	4	5	0.80
Iowa	2003–2009	6	7	0.86
Louisiana	2003–2011	0	9	0.00
Michigan	2005–2009	3	5	0.60
Minnesota	2003–2009	13	7	1.86
Missouri	2003–2005	2	3	0.67
North Carolina	2003–2011	6	9	0.67
New Jersey	2003–2005, 2007	1	4	0.25
New York	2003–2011	67	9	7.44
Oregon	2003–2011	1	9	0.11
Texas	2003–2009	1	7	0.14
Utah	2003–2011	3	9	0.33
Washington	2003–2009	4	7	0.57
Wisconsin	2003–2011	23	9	2.56

* Average.
